# Nutritional physiology and ecology of wildlife in a changing world

**DOI:** 10.1093/conphys/cox030

**Published:** 2017-05-22

**Authors:** Kim Birnie-Gauvin, Kathryn S. Peiman, David Raubenheimer, Steven J. Cooke

**Affiliations:** 1 Fish Ecology and Conservation Physiology Laboratory, Department of Biology and Institute of Environmental Science, Carleton University, 1125 Colonel By Drive, Ottawa, Canada ON K1S 5B6; 2 DTU AQUA, National Institute of Aquatic Resources, Section for Freshwater Fisheries Ecology, Technical University of Denmark, Vejlsøvej 39, 8600 Silkeborg, Denmark; 3 Faculty of Veterinary Science, The University of Sydney, Regimental Drive, Camperdown, NSW 2050, Australia

**Keywords:** Conservation, diet, human-caused environmental changes, nutritional ecology

## Abstract

Over the last century, humans have modified landscapes, generated pollution and provided opportunities for exotic species to invade areas where they did not evolve. In addition, humans now interact with animals in a growing number of ways (e.g. ecotourism). As a result, the quality (i.e. nutrient composition) and quantity (i.e. food abundance) of dietary items consumed by wildlife have, in many cases, changed. We present representative examples of the extent to which vertebrate foraging behaviour, food availability (quantity and quality) and digestive physiology have been modified due to human-induced environmental changes and human activities. We find that these effects can be quite extensive, especially as a result of pollution and human-provisioned food sources (despite good intentions). We also discuss the role of nutrition in conservation practices, from the perspective of both *in situ* and *ex situ* conservation. Though we find that the changes in the nutritional ecology and physiology of wildlife due to human alterations are typically negative and largely involve impacts on foraging behaviour and food availability, the extent to which these will affect the fitness of organisms and result in evolutionary changes is not clearly understood, and requires further investigation.

## Teaser

Humans have modified planet Earth extensively, with impacts ranging from reduced habitat availability to warming temperatures. Here we provide an overview of how humans have modified the nutritional physiology and ecology of wild organisms, and how nutrition is vital to successful conservation practices.

## Introduction

In the last century, humans have modified the global landscape to accommodate the growing human population (Vitousek *et al.*, 1997). Previously pristine landscapes, riverscapes and seascapes have been transformed as a result of agriculture, urbanization, resource extraction (e.g. mines, forestry, fishing), energy production (e.g. hydropower, fossil fuels), military activity, and other human developments and activities (Marzluff *et al.*, 2001; Foley *et al.*, 2005; Dudgeon *et al.*, 2006; Kennish, 2002; Crain *et al.*, 2009). The accumulation of human-induced changes has modified ecosystems to the point where human activities are now considered the primary driver of global change (Vitousek *et al.*, 1997; Sanderson *et al.*, 2002) and it is proposed that we have entered a new epoch called the Anthropocene (Crutzen, 2006). As a result, humans have changed the environment in which wild animals live, including the abundance and quality of food items which has implications for animal health, reproduction and survival (Acevedo-Whitehouse and Duffus, 2009).

In recent years, two fields of nutrition have grown significantly (Frost *et al.*, 2014). Nutritional ecology investigates the relationships among diet, digestive physiology and feeding behaviour (Foley and Cork, 1992; Raubenheimer *et al.*, 2009), while nutritional physiology focuses on the subset of relationships related to the intake and assimilation of food items. Here we focus on how food choices and digestion are affected by the abundance (quantitative limitation) and composition (qualitative limitation) of the foods available in a particular environment (Lambert, 2007; see Table 1 for commonly used tools to evaluate nutrition in animals). These fields have generated many insights that have made them a cornerstone for understanding the mechanisms that link ecological patterns and processes to animal phenotypes (Raubenheimer *et al.*, 2009; Karasov *et al.*, 2011; Simpson and Raubenheimer, 2012). They are also important in understanding the constraints that nutrition may impose on locomotion, activity patterns, demography and population dynamics (Foley and Cork, 1992; Raubenheimer *et al.*, 2015).

**Table 1:** Brief summary of the common methods used to study nutrition in animals, with a description of the advantages and disadvantages for each

Method	Pros	Cons
Gut sampling	Provides insight into specific ingested prey items, nutrient intake, energetic intake	Insight into short-term diet only; Ingested organisms may be mistaken during identification; Typically requires lethal sampling or high levels of induced stress (stomach lavage) but new options with DNA assessment of gut materials are being developed
Tissue sampling	Provides insight into macromolecules	Often requires lethal sampling
Faecal analysis	Non-invasive, does not require capture, or lethal sampling	Difficult to match faeces to a particular individual if behaviour is important to the study; Soft-bodied prey often not identifiable
Stable isotopes	Provides insight into short and long-term diet	Costly; Does not provide information on specific ingested foods
Direct behavioural observations	First hand observations of what foods are ingested	Very time-consuming; Human presence can sometimes alter feeding behaviour
Bio-logging or biotelemetry	Provides information on the spatial and temporal patterns of foraging behaviour and food intake	Data may take time to examine; Electronic tags tend to be expensive

Currently there is no cohesive framework that posits how human-induced environmental change influences the nutrition of wild vertebrates. Such a framework would be particularly useful for developing testable hypotheses concerning the future implications of such alterations. In this paper, we present an overview of the ways in which humans have altered the environment (climate change, pollution, habitat loss/fragmentation, invasive species, human disturbances and provisioned food sources), and consider how each of these modifications may affect the acquisition, availability (quality and quantity) and digestion of food for vertebrates. We additionally present representative examples that demonstrate the extent to which humans can impact the nutrition of wild vertebrates. We address how nutrition has been used in the context of *in situ* and *ex situ* conservation. We focus on vertebrates given their imperilled status (Sala *et al.*, 2000), interest from conservation practitioners and policy makers (Redford *et al.*, 2011) and because relative to most invertebrates, the basic biology, natural history and nutritional ecology of vertebrates are well studied (see Donaldson *et al.*, 2016), an understanding owing in large part to them being commonly held in zoos and aquaria (Conde *et al.*, 2011).

## Human-induced environmental changes and their effects on nutrition

Humans have altered the planet in many ways including through climate change, pollution, habitat alteration and the introduction/translocation of new species (reviewed in Vitousek *et al.*, 1997). What does this mean to animals in terms of nutrition?

### Climate change

In today’s warming world, shifts in moisture, carbon dioxide, temperature and solar radiation are pervasive (IPCC, 2013), and these changes will directly and indirectly affect animal performance by influencing the composition of their food (Post and Stenseth, 1999; Kearney *et al.*, 2013; Rosenblatt and Schmitz, 2016). These predicted changes are far-reaching and complex, and their interactions among trophic levels are still poorly understood. For example, changes to primary producers involve both quality and abundance: increased temperature may lead to increased stratification of the water column in parts of the ocean, creating nutrient limitation and changing the dominant species of phytoplankton with unknown effects on higher trophic levels (Beardall *et al.*, 2009); and Lake Tanganyika in Africa has already undergone decreases in phytoplankton productivity due to increased stratification from a combination of increased temperature and decreased wind velocity, leading to a decline in pelagic fishes (O’Reilly *et al.*, 2003). On land, elevated CO_2_ typically causes an increase in tissue carbon in plants, accompanied by decreases in nitrogen (Cotrufo *et al.*, 1998), phosphorus (Gifford *et al.*, 2000) and other elements (Loladze, 2002), including protein (Robinson *et al.*, 2012). When feeding on these plants, insects had decreased growth but increased consumption (Robinson *et al.*, 2012) indicating that the nutritional quality of the plants had diminished. Food protein content is often associated with animal performance, and so a decrease in the ratio of protein energy to non-protein energy (i.e. protein vs carbohydrates and lipids; Raubenheimer *et al.*, 2014) will reduce the quality of plant foods available to wildlife (Zvereva and Kozlov, 2006). Plants may also undergo increases in toxic secondary compounds under increased temperatures which may affect the ability of herbivores to meet their nutritional requirements (Moore *et al.*, 2015). Most of these changes have been documented for the primary producers themselves, with much less research on the subsequent nutritional effects on their consumers.

Climate change can also directly impact secondary and tertiary consumers in several ways. One way it does this is to cause further physiological impairments when combined with decreased nutritional intake (Robbins, 1993; Murray *et al.*, 2006). For example, when prey is depleted and individuals catabolize their fat reserves, lipophilic toxins such as PCBs can be released (Jepson *et al.*, 2016). In herbivores, body condition influences how individuals choose locations of high forage quality versus tolerable thermal stress (Long *et al.*, 2014). These trade-offs may be particularly severe during the energy-intensive time of reproduction (egg or embryo development in females, pregnancy or lactation in female mammals, and/or parental care) (Lewis, 1993; Ashworth *et al.*, 2009) or be more intense in animals with certain reproductive strategies (e.g. income versus capital breeders; Costa, 2012). For example, nest success in parental male smallmouth bass (*Micropterus dolomieu*) is affected by both body size and climatic indices (Suski and Ridgway, 2007). Because the activity and development of many insects depend on climatic conditions (Burles *et al.*, 2009), food availability for insectivores will likely be highly affected by climate change (Sherwin *et al.*, 2013; Berzitis *et al.*, 2017). As lower trophic levels can adapt their phenologies in response to climate change faster than their consumers can, shifts in peak abundance of food may no longer align with periods of vertebrate offspring growth and development (Davies and Deviche, 2014). Additionally, when nutrient dispersers such as bats are affected by climate change, this will presumably affect the extent to which nutrients will be dispersed over the landscape, potentially having important repercussions on other animals, though this link has not yet been investigated.

### Pollution

Changes in animal behaviour can occur at concentrations of chemicals lower than can cause mortality (Little and Finger, 1990) and may affect foraging decisions (Scott and Sloman, 2004; Vaughan *et al.*, 1996). Environments that are heavily contaminated by metals have a reduced abundance and diversity of many terrestrial insects (reviewed in Heliövaara and Väisänen, 1990), which can affect the breeding performance of insectivorous birds (Eeva *et al.*, 1997). Birds exposed to metal pollutants also showed decreased appetite (Di Giulio and Scanlon, 1984) and paper mill effluents interfere with digestive enzymes in fish (Temmink *et al.*, 1989). Thus, pollutants potentially have accumulating effects: they reduce the food supply available, decrease interest in the available food and reduce digestion of food that is consumed. Predators are especially vulnerable because many compounds undergo bioaccumulation, exposing animals higher in the food chain to elevated levels of pollutants (Walker, 1990).

In streams, lakes and estuaries, water can become turbid through a variety of processes (Smith, 1990). This results in changes in the abundance and diversity of primary producers (Smith, 2003) and affects the ability of consumers to detect prey (Utne-Palm, 2002; Chivers *et al.*, 2013; Chapman *et al.*, 2014). In general, turbidity is predicted to affect piscivorous fish more than planktivorous fish due to differences in attack distances (De Robertis *et al.*, 2003) but turbidity also changes the behaviour of prey fish due to decreased risk of predation (Pangle *et al.*, 2012). For example, under turbid conditions perch (*Perca fluviatilis*) had reduced capture rates of benthic prey and slower growth rates (Ljunggren and Sandstrom, 2007), and brown trout (*Salmo trutta*) consumed a lower diversity and abundance of benthic prey and had lower condition (Stuart-Smith *et al.*, 2004). In planktivores, bluegill sunfish (*Lepomis macrochirus*) showed reduced feeding rates under increased turbidity (Gardner, 1981); larval herring (*Clupea harengus pallasi*) increased feeding on plankton at low turbidity but decreased feeding at high turbidity (Boehlert and Morgan, 1985); and perch captured fewer zooplankton with increasing turbidity while no effect was seen in roach (*Rutilus rutilus*) (Nurminen *et al.*, 2010). Turbidity can also change prey selection. Piscivorous, benthivorous and planktivorous species have all showed shifts in prey composition in turbid environments (Hecht, 1992; Stuart-Smith *et al.*, 2004; Shoup and Wahl., 2009; Johansen and Jones, 2013). Turbidity has clear effects on foraging behaviour and diet quantity and composition, but the fitness effects of these changes are not known.

Plastic debris accounts for 60–80% of the total debris in marine environments, coming from accidental equipment loss, careless handling (e.g. land-based trash washing to sea) and littering (reviewed in Derraik, 2002; Galgani *et al.*, 2015) but freshwater habitats also have large plastic debris loads (Wagner *et al.*, 2014). Such pollution can greatly reduce the quantity of food that organisms can eat through a reduced ability to move (entanglement or injury) or a blockage of the digestive system (ingested debris) (Quayle, 1992; Laist, 1997; Wilcox *et al.*, 2015; Holland *et al.*, 2016). Ingested debris can result in a reduction of the area available for nutrient absorption in animals ranging from sea turtles (McCauley and Bjorndal, 1999; Schuyler *et al.*, 2016) to stickleback (Katzenberger, 2015) to beachhoppers (*Platorchestia smithi*) (Tosetto *et al.*, 2016) and can create a physical blockage of the digestive tract (Danner *et al.*, 2009) which impedes further food intake and digestion. The accumulation of debris on the seafloor (Galgani *et al.*, 2015) may also reduce the productivity and species composition of plants and prey items (reviewed in Kuhn *et al.*, 2015). Overall, plastics more commonly affect the ability of individuals to eat sufficient amounts of food rather than affecting the quality of food available, but the impact of plastics on trophic linkages has been identified as a global research priority (Vegter *et al.*, 2014).

### Habitat quantity and quality

Humans have altered landscapes extensively, causing habitat loss and fragmentation, which leads to changes in the physical environment and biogeography of plants and animals (reviewed in Saunders *et al.*, 1991). Because some species are restricted in the habitats they can occupy or type of food they can consume, these landscape modifications can severely reduce their population sizes, therefore restricting the abundance of prey for their predators. For example, habitat fragmentation can cause a decline in pollination and seed set (Rathcke and Jules, 1993), thereby reducing the abundance of certain plant species and presumably affecting the herbivores that feed on them. Additionally, when animals choose habitats based on cues that are no longer appropriate, they experience an ecological trap and may undergo population declines (Schlaepfer *et al.*, 2002). In general, specialist species are likely to be affected by habitat modification to a greater extent than generalist species (Devictor *et al.*, 2008). However, some species are able to switch to foods that are more readily available when their preferred food source is scarce (Felton *et al.*, 2009), suggesting that behavioural plasticity is an important factor to consider in order to fully understand the impacts of habitat alteration on animal nutrition (Tuomainen and Candolin, 2011). Other modifications to the landscape can also affect the nutrition of wildlife. For example, fires are controlled in many areas, but burning can increase the quality of grass species for herbivores (Hobbs and Spowart, 1984). Humans have also modified the land to accommodate infrastructures in order to meet anthropogenic needs (e.g. oil well sites, hydroelectric dams, roads). Such infrastructure reduces population size by replacing natural habitat and causing animals to avoid those areas (e.g. reindeer: Nellemann *et al.*, 2003; bears: Gibeau *et al.*, 2002; amphibians; Hamer and McDonnell, 2008; birds and mammals: Benitez-Lopez *et al.*, 2010), consequently reducing food abundance for their predators.

Some regions of the world have been depleted of their native vegetation by 93% and this has been replaced by agricultural land (Saunders *et al.*, 1990), thereby providing crops as an alternative food source. Some crops are nutritionally attractive to wild animals and provide both energy (Sukumar, 1990; Riley *et al.*, 2013; McLennan and Ganzhorn, 2017) and minerals (Rode *et al.*, 2006a). However, the effects on the health of these species are poorly studied. In contrast, the availability of grain crops in the winter for several species of geese has provided an excellent food source (Gates *et al.*, 2001; Ely and Raveling, 2011) though some crop types are deficient in nutrients (Alisauskas *et al.*, 1988). Thus the effects of replacing native vegetation with alternative food sources are still not known for most herbivores.

### Invasive species

Human-caused habitat disturbance has been associated with an increased likelihood of invasion of communities by non-native species (Hobbs and Huenneke, 1992), such as large oil well sites which increase the presence of non-native plants (Preston, 2015). Some now invasive species were even purposely planted as food for wildlife (Kaufman and Kaufman, 2007), even though native plants are often nutritionally better for herbivores than introduced species (Applegate, 2015). Biological invasions contribute to the worldwide decline in biodiversity by changing the abundance and richness of communities (Clavero and García-Berthou, 2005). This alters prey abundance, but the direction of this effect will depend on whether invaders affect common or rare native species (Powell *et al.*, 2011) and whether herbivores and predators prefer to consume native or introduced species (Morrison and Hay, 2011; Jaworski *et al.*, 2013). Introduced species can also affect the diet quality of their consumers, but this effect will depend on how the ratio of nutrients and secondary compounds differs between native and introduced prey (Maerz *et al.*, 2010).

Introduced species can have diverse effects on species interactions. A famous example of a successful invasive species is the Eurasian zebra mussel (*Dreissena polymorpha*). Zebra mussels modify the concentration of nutrients and the community of algae in whole ecosystems (Caraco *et al.*, 1997) thus affecting the diet of native species through changing the availability of alternative food (Gonzalez and Downing, 1999), and through consumption as a direct food source that for some species provides less energy than normal prey (Watzin *et al.*, 2008). In Australia, toxic cane toads (*Bufo marinus*) were introduced to deal with plant pests, but their presence has had many unintended consequences. For example, northern trout gudgeon (*Mogurnda mogurnda*) exposed to cane toad tadpoles showed reduced rate of consumption of native tadpoles (Nelson *et al.*, 2010) and adult cane toads reduce the activity of native frogs during foraging (Mayer *et al.*, 2015). Introduced benthivorous fish, such as goldfish (*Carassius auratus*) and common carp (*Cyprinus carpio*), increase water turbidity through the mechanical actions of foraging, thus affecting the foraging success of other aquatic species (see ‘Pollution’ section) (Richardson *et al.*, 1995; Zembrano *et al.*, 2001). However, there has been a lack of study focused on the nutritional effects on native animals beyond simple consumption, and none linking these effects to fitness.

### Anthropogenic disturbances

Human disturbance can modify feeding strategies through increased nocturnal illumination and acoustic disturbances. Natural lighting cycles affect foraging in a wide variety of species (reviewed in Navara and Nelson, 2007) and so it should be no surprise that artificial lighting changes these behaviours, especially as it can exceed the intensity of any natural lunar phase (Cinzano *et al.*, 2001). Both prey and predators are affected by artificial light. Insects are readily attracted to nocturnal lights, and this is changing not only the abundance but also the species composition of this prey base (Davies *et al.*, 2012). Some prey reduce foraging under lights (Kotler, 1984; Contor and Griffith, 1995; Brown *et al.*, 1998; Baker and Richardson, 2006) while others increase it (Biebouw and Blumstein, 2003), changes often linked to increased predation risk under illumination (Rich and Longcore, 2013). Similarly, night lighting may impair the vision of some predators (Buchanan, 1993) while others are more active and use the increased visibility (Yurk and Trites, 2000; Rich and Longcore, 2013) which may change their distribution in the environment (Montevecchi, 2006). However, when the light itself mimics a foraging cue, individuals may not possess the flexibility to change their behaviour (Schlaepfer *et al.*, 2002). For example, juveniles of many seabird species are drawn to lights, possibly because they resemble their bioluminescent prey (Montevecchi, 2006)—a clearly maladaptive response. In general, the severity of the effects of artificial illumination will depend on the trade-off between predation, foraging and competition, whether the species are naturally nocturnal or diurnal, and whether these new cues trigger previously adaptive responses.

Acoustic disturbance has increased drastically over the past century, affecting communication in urban populations (Birnie-Gauvin *et al.*, 2016). Anthropogenic noise can have similar effects to artificial lighting in that it may hinder an individual’s ability to identify prey and/or predators, or lead to chronic stress, which may in turn lead to decreased foraging efficiency and lower reproductive success (National Research Council, 2005; Schroeder *et al.*, 2012: Meillere *et al.*, 2015: Shannon *et al.*, 2015). This form of feeding disturbance is especially detrimental to animals that rely on acoustic cues to locate food items. For example, sonar-using greater mouse-eared bats (*Myotis myotis*) spend less time foraging when exposed to traffic noise (Jones, 2008). However, some species have the ability to cope with noise pollution. For example, the foraging behaviour (i.e. diving frequency) of mysticete whales (*Balaenoptera physalus* and *B. musculus*) was largely unaffected by low frequency sounds which are typical of cargo ships and oil development infrastructure (Croll *et al.*, 2001). In fact, whale behaviour appeared to be more closely related to prey abundance than to acoustic disturbance (Croll *et al.*, 2001). When noise causes individuals to shift attention, foraging often suffers. For example, noise led chaffinches (*Fringilla coelebs*) to increase vigilance (scanning for predators) and decrease food intake (Quinn *et al.*, 2006), and caused decreased foraging efficiency in three-spined stickleback (*Gasterosteus aculeatus*) (Purser and Radford, 2011). However, if specialists are also more efficient at foraging, additional time dedicated to detecting predators may be more costly to generalist species (Chan and Blumstein, 2011). The contrasting results from studies that investigate the effects of noise on feeding behaviour suggests that depending on the feeding nature of organisms, they may be affected differently and to varying degree. Many reviews have suggested that foraging is affected by noise (Kight and Swaddle, 2011; Francis and Barber, 2013), but few studies have made direct links to nutrition.

Another important form of disturbance is the very presence of humans, which is presumably the most direct form of anthropogenic disturbance for wild organisms and generally results in an energy cost (Houston *et al.*, 2012). This may come in the form of hunting, horseback riding, biking, hiking, camping, swimming, fishing, skiing, photographers, or observers (Cole and Knight, 1991; Boyle and Samson, 1985; Knight and Gutzwiller, 1995; Hammitt *et al.*, 2015). The effects of such recreational activities on nutrition have seldom been investigated, but behaviour can be highly affected by human presence. For example, the presence of observers near the territories of European oystercatchers (*Haematopus ostralegus*) led to less time spent foraging and reduced food intake for the parents, and decreased the proportion of food allocated to the chicks (Verhulst *et al.*, 2001). In marsh harriers (*Circus aeruginosus*), disturbance by fisherman, passers-by, dogs, and vehicles also resulted in lower food provisioning and higher nutritional stress in chicks (Fernández and Azkona, 1993). However, brown bears (*Ursus arctos*) showed minimal effects of human presence as they altered their behaviour to maintain food intake and body condition (Rode *et al.*, 2006c, 2007). Yet the same species of bear decreased their foraging activity and fed on berries of poorer quality when hunting risk was high (Hertel *et al.*, 2016). When endangered Amur tigers (*Panthera tigris altaica*) were disturbed, they often abandoned kills, spent less time at the kill when they stayed and consumed less meat (Kerley *et al.*, 2002). Elk (*Cervus elaphus*) fled in response to skiers, often moving upslope to areas with poorer quality vegetation (Frances Cassirer *et al.*, 1992). Bald eagles (*Haliaetus leucocephalus*) rarely fed at salmon carcasses when disturbed while glaucous-winged gulls (*Larus glaucescens*) fed more, indicating gulls were more wary of the dominant heterospecific than of people (Skagen *et al.*, 1991). Disturbance also led to changes in temporal feeding activity of bald eagles, crows and ravens (Knight *et al.*, 1991). Responses to people may also differ between the sexes. Female brown bears with young prioritize avoidance of male bears over avoidance of humans, while male site use was linked to prey availability (Rode *et al.*, 2006b). The presence of people often results in behavioural modifications in feeding activity or location that may result in poorer body condition and lower reproductive success in animals that are sensitive to this presence.

### Human-provisioned food sources

In urban areas, humans often provide a source of food for many wild animals, both inadvertently (e.g. through garbage) or on purpose (e.g. bird seeds in the backyard; Murray *et al.*, 2016). In most industrialized countries, these foods have a high level of predictability both spatially and temporally (Chamberlain *et al.*, 2005; Oro *et al.*, 2013). Such food provisioning may affect food webs and communities, changing competitive and predator-prey interactions and nutrient transfer processes (reviewed in Oro *et al.*, 2013), primarily due to ease of access in comparison to natural food sources (Bartumeus *et al.*, 2010) which reduces time spent foraging (Orams, 2002).

Unintentional food provisioning usually involves refuse sites (dumps, middens, harvest discards, etc.). Many cosmopolitan opportunistic species such as gulls, rats and foxes have benefited greatly from these food subsidies, showing improved body condition and reduced susceptibility to pathogens (reviewed in Carey *et al.*, 2012; Oro *et al.*, 2013). Vervet monkeys (*Chlorocebus pygerythrus*) spent less time foraging and had higher reproduction but also increased aggression while feeding on garbage (Lee *et al.*, 1986), while olive baboons (*Papio anubis*) with access to garbage also spent less time foraging and had higher body condition and lower levels of parasite infection than naturally-foraging groups (Eley *et al.*, 1989). In other cases, food provisioning is not beneficial. For example, fisheries bycatch provides seabirds with access to prey that have a lower energetic content than their normal pelagic prey (Grémillet *et al.*, 2008). During the non-breeding season seabirds can use bycatch and still meet their own nutritional needs, but when breeding commences females need to consume pelagic prey due to the energetic requirements of egg formation (Louzao *et al.*, 2006; Navarro *et al.*, 2009) and chicks fed on bycatch have lower growth rates and survival (Grémillet *et al.*, 2008). Unintentional provisioning may also include cultivated fruit trees, compost and dropped bird seed, all of which are highly attractive to urban wildlife (reviewed in Murray *et al.*, 2015). These low-protein but easily accessible foods may either cause poor health or be used by animals already in poor health, increasing the likelihood of human-wildlife conflicts (Murray *et al.*, 2015). Human food sources can also increase interactions among wildlife. For example, Steller’s jay (*Cyanocitta stelleri*) utilizes anthropogenic food at campsites, and though the effects on the jay’s nutrition are not known, access to this food source may result in increased predation on the endangered marbled murrelet (*Brachyramphus marmoratus*) (Goldenberg, 2013). When food left at campsites attracts flocks of carnivores and omnivores, small-bodied herbivores may be excluded from the area (Densmore and French, 2005). Thus the extent, timing and quality of human-provisioned resources will determine the effects of using this alternative prey.

Wildlife tourism is an important source of income for many countries (Braithwaite, 2001) and can be a motivation for intentional feeding (reviewed in Orams, 2002). However, this form of interaction can be highly detrimental to wildlife (Murray *et al.*, 2016). For example, both stingrays (Semeniuk *et al.*, 2007) and iguanas (Knapp *et al.*, 2013) fed by tourists show poorer indicators of adequate nutrition than those eating natural food. Moreover, interactions at food sources can lead to increased risk of injury for animals, as is observed in chacma baboons (*Papio ursinus*) where these injuries also hindered their foraging efficiency (Beamish, 2009). The feeding of wildlife can also cause an aggregation of individuals at feeding sites (Newsome and Rodger, 2008), potentially reducing food intake per individual through competition (Raman, 1996). Even backyard feeding of birds can affect subsequent reproduction (Ruffino *et al.*, 2014) as provisioned food is often calorie-rich but nutrient-poor (Plummer *et al.*, 2013). Provisioned food may even have unpredictable effects on nutrition when it interacts with other components of the diet. For example, white-tailed deer supplemented with hay and corn consumed less digestible energy in areas where they also consumed lichen which reduces feed retention times (Page and Underwood, 2006). Humans enjoy being in close contact with animals, but when this involves feeding wildlife, the health of the wildlife is often of secondary importance. Some of these negative effects of ecotourism may be overcome by focusing on animals that possess sufficient behavioural plasticity to eliminate the effects of humans on an individual’s spatiotemporal resource use (e.g. brown bears: Rode *et al.*, 2007). Additionally, when ecotourism is designed to reduce negative impacts on wildlife and is also used as a source of education about their proper feeding, everyone benefits (Ballantyne *et al.*, 2009).

The degree to which food supplementation has long-term effects on populations remains largely unknown. There are few long-term studies of the effects of supplemented feeding on nutrition in wildlife (Orams, 2002). The evolutionary consequences have so far been virtually ignored, even though it has been hypothesized that in cases where the more aggressive individuals obtain the most food and thus leave more offspring, supplemental feeding can be a source of selection and change the phenotypes in a population (Moribe, 2000). Nonetheless, when the provisioning of additional food items has benefits such as higher survival (Orams, 2002), these short-term gains may be important enough to offset the possible effects on population dynamics. This concept has recently been extended to include the use of carcass provisioning as a conservation strategy to enhance survival for scavenger species (Fielding *et al.*, 2014).

## Nutrition and *in situ* conservation

**Figure 1:**
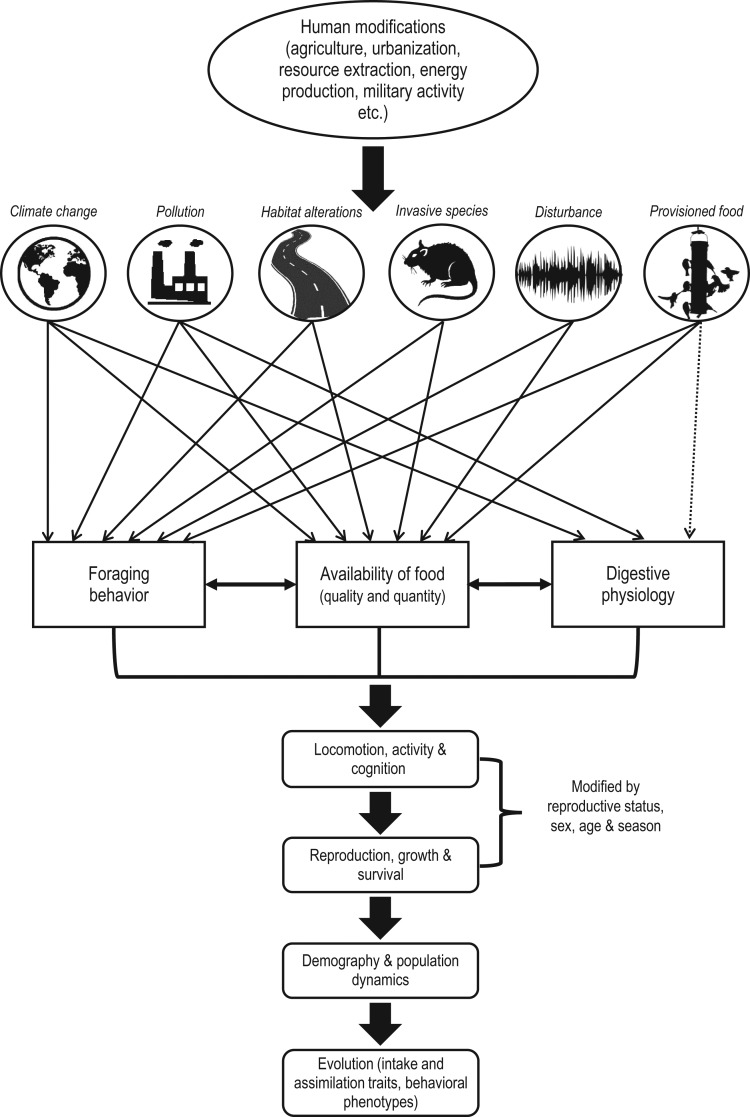
Anthropogenic effects on components of animal nutrition. Human presence has altered the environment. Here, we identify how these human modifications (climate change, pollution, invasive species, habitat alterations, disturbance and human-provisioned food) affect aspects of nutrition through effects on foraging behaviour, food availability and digestive physiology (solid black arrows represent links already established in the literature; dotted arrows represent hypothetical links). Depending on how these three aspects of nutrition are altered, locomotion, activity and cognition may change, affecting reproduction, growth and survival. These may in turn affect demography and population dynamics, which may affect evolutionary processes.

*In situ* conservation (e.g. habitat restoration, supplemental feeding) aims to manage and protect species in natural habitats (Possiel *et al.*, 1995). In the context of nutrition, this requires balancing foraging behaviours and food availability (which are affected by all six categories of human-caused modifications; Fig. 1) with nutritional physiology. This may involve studying foraging ecology, measuring the nutritional composition of foods, providing non-naturally occurring food, and investigating these impacts on digestive physiology to ensure sufficient energy and nutrient intake (Hobbs and Harris, 2001; Hobbs *et al.*, 2009). For example, it was necessary to supplement the endangered hihi (*Notiomnystis cincta*) with carbohydrates to increase reproductive success (Castro *et al.*, 2003), and knowledge of foraging behaviours and nutrient requirements of the vulnerable Tonkean macaques (*Macaca tonkeana*) can help reduce damaging crop raiding behaviours (Riley *et al.*, 2013). Detailed studies of wild populations are often necessary to know what forage species are preferred (used versus available: Johnson, 1980). They may have to be long-term to account for seasonal (e.g. Karachle and Stergiou, 2008; Adeola *et al.*, 2014) or inter-annual (Esque, 1994) variation in prey consumption, and they may have to measure many individuals and multiple populations as the level of individual dietary specialization can vary with resource availability (Bolnick *et al.*, 2002), individual mechanisms to deal with changing food availability vary with sex and condition (Martin, 1987), and food preference can be under genetic control and locally adapted (Sotka, 2003). Actual measures of nutrition are often invasive, and non-invasive alternatives are still lacking validation for many wild species (Murray *et al.*, 2016). Thus, measuring food intake and diet composition for wild animals is a difficult task, but there are many techniques that make addressing these questions possible (Cooke *et al.*, 2004; Robbins *et al.*, 2004; Servello *et al.*, 2005; Andrews *et al.*, 2008; Rothman *et al.*, 2012; Machovsky-Capuska *et al.*, 2016; see Table 1).

An example involves the desert tortoise (*Gopherus agassizii*), which was put under the Endangered Species Act in 1989 due to huge population declines (U.S. Fish and Wildlife Service, 1994). The threats to the desert tortoise were considered to be mostly physiological, of which many could be attributed (directly or indirectly) to nutrition (Tracy *et al.*, 2006). The presence of domestic grazers, the occurrence of fires and the invasion of weedy plants—all of which are largely caused by humans—contributed to their nutritional deficiencies by reducing plant diversity (U.S. Fish and Wildlife Service, 1994). Each tortoise obtained approximately 90% of their diet from 5 species of plants, but the specific species eaten differed across individuals resulting in more than 30 species of plants consumed at the population level (Tracy *et al.*, 2006). The mechanisms causing this were complex, and mainly involved choice of plants with high digestible energy (used versus available), and individual encounters with specific plant species early in the season (switching foods incurs a cost when gut microbes are specific to the plants consumed), suggesting that a variety of species should be made available to tortoises in the context of *in situ* conservation to fulfil individual nutrition needs (Tracy *et al.*, 2006). Inadequate dietary intake caused by low species diversity can induce stress and lead to compromised immunity and increased susceptibility to disease, which in the case of the desert tortoise has had severe impacts on population densities, providing evidence for the importance of nutrition in conservation biology; Box 2.

## Nutrition and *ex situ* conservation

While *in situ* conservation approaches have been considered a legal and institutional priority by the Convention on Biological Diversity (www.cbd.int), it is increasingly apparent that the importance of *ex situ* conservation is growing, as extinction rates continue to rise and are exacerbated by climate change (Pritchard *et al.*, 2012). *Ex situ* conservation aims to conserve species in captivity and relies on facilities that hold plants and animals such as zoos, aquaria and botanical gardens, and even private breeders. The knowledge gained from these facilities can also be used to support conservation efforts. When inadequate diets in captivity lead to an individual’s death or failure to reproduce, there is increasing pressure to collect more individuals from the wild. For example, many species of parrots and iguanas are popular pets. However, these pets are often fed nutritionally inadequate diets, leading to death via malnutrition or increased susceptibility to disease and more animals collected illegally from the wild (Dohoghue, 1994; Schlaepfer *et al.*, 2005; Weston and Memon, 2009). If these owners were made aware of proper nutrition for these birds, harvest of wild populations would decrease.

The importance of meeting nutritional requirements to conserve and manage endangered and at-risk species should not be understated (Oftedal and Allen, 1996; see Box 3). Food quantity continues to be the primary focus in zoological parks, despite the recognition that food quality plays a huge role in maintaining animal health and reproductive potential (see Box 1). For example, in captive ruminants, browsers have a higher nutrition-related mortality than grazers because browsers are fed a type of roughage that is not very similar to their natural foods, resulting in too little roughage ingested compared to seeds/grains and causing digestion issues (Müller *et al.*, 2010). Providing foods and food combinations of adequate quality is a more difficult task than food quantity, the latter which can be addressed by simply providing more known suitable foods. In a captive breeding project, green iguana (*Iguana iguana*) hatchlings and juveniles grew more rapidly when fed diets high in protein than when fed lower protein diets (Allen *et al.*, 1989). High growth rates are considered important for young iguanas as predation risks are high and thus individual size determined the age at which these iguanas could be released into the wild (Oftedal and Allen, 1996). In captive mule deer (*Odocoileus hemionus*), diets supplemented with feed concentrates, oats and barley resulted in increased body mass and antler size, as well as earlier breeding and a decrease in fawn mortality (Robinette *et al.*, 1973). Following this increased food intake, the productivity of this captive herd surpassed that of wild populations (Robinette *et al.*, 1973).

Box 1. **The importance of nutrition for animals**

Adequate dietary intake (both calories and nutrients) is essential to the growth and reproductive success of vertebrates. In fact, the physiological component of reproduction and sexual behaviour is extremely sensitive to the intake of metabolic fuels (Wade *et al.*, 1996; Allen and Ullrey, 2004; Parker *et al.*, 2009). Organisms will forego reproduction if they do not have the energetic resources to invest in gonadal development or reproductive activities. However, calories alone are insufficient for the maintenance of health, growth (somatic or reproductive) and other routine functions such as cognition. For example, mammals require proper nutrients for successful parturition and the production of colostrum and milk, while birds require calcium to make eggshells (Robbins, 1993). Proteins and amino acids are crucial for proper organ development (Welham-Simon *et al.*, 2002) and egg production (Ramsay and Houston, 1998). Fatty acids are essential for brain development and neurogenesis (Schiefermeier and Yavin, 2002), as well as for components of spermatozoa (Surai *et al.*, 2000). Minerals and vitamins are also an important aspect of nutrient intake. For example, a lack of dietary selenium can impair reproductive performance (Cantor and Scott, 1974), while zinc deficiency is linked to testicular underdevelopment (Martin *et al.*, 1994). Vitamin A is a crucial micronutrient for proper eye development, vision and cellular differentiation (National Research Council, 1995), and vitamins E and C are important for oxidative homeostasis (Castellini *et al.*, 2000). In addition, it has long been recognized that diet plays an essential role in maintaining immunity against diseases (Lall and Olivier, 1993). For example, megadoses of vitamin C have been shown to improve antibody response and survival following infection in the channel catfish (*Ictalurus punctatus*; Li and Lovell, 1985; Liu *et al.*, 1989). Diet also affects cognitive processes: lipid-poor diets decrease the ability of kittiwakes (*Rissa brevirostris*) to learn the location of food (Kitaysky *et al.*, 2006). Thus, macronutrients and micronutrients play essential roles in the proper development of animals, from embryo to reproductive adult.

Box 2. **Nutrition in *in situ* conservation: the tiger**

Tigers (*Panthera tigris*) are a globally endangered species that have suffered huge population losses as a result of human presence (Chundawat *et al.*, 2012). However, in Nepal, conservation efforts have resulted in the tiger population increasing by 63% in recent years (Government of Nepal, 2013; Aryal *et al.*, 2016). Yet the prey biomass within currently protected areas may be insufficient to provide food for the projected increased tiger population (Aryal *et al.*, 2016). It has been suggested that programs should be implemented to increase prey populations *in situ* to continue conservation efforts and restore tiger populations. (*Image by Martin Harvey, World Wildlife Fund*)

**Figure:**
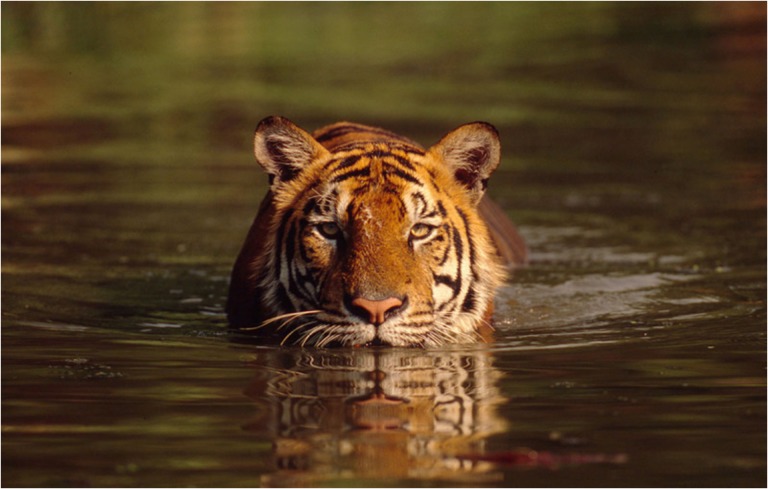

Box 3. **Nutrition in *ex situ* conservation: the kakapo**

The kakapo (*Strogops habroptilus*) is a large, flightless parrot endemic to New Zealand. It was put on the critically endangered list in 1989, largely due to catastrophic population declines caused by introduced mammalian predators (Williams, 1956: Powlesland and Lloyd, 1994). The kakapo only breed in years during which podocarp trees produce abundant fruit, which occurs every 2–6 years (Powlesland and Lloyd, 1994; Cockrem, 2006). When supplemented with specially formulated pellets that contained protein, micronutrients, mineral supplements and amino acids, females produced larger clutches but did not change nesting frequency, suggesting that podocarp fruiting is the cue for breeding while the number of eggs is limited by nutritional quality rather than energetic content (Houston *et al.*, 2007). Hand-rearing of chicks using artificial foods now plays a critical role in the management of this critically endangered species (Waite *et al.*, 2013). (*Image by Milena Scott*)

**Figure:**
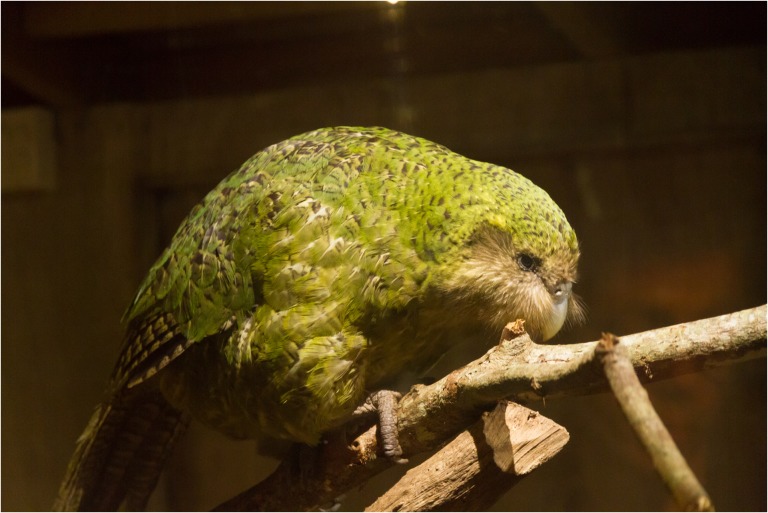

## Conclusion and research needs

Nutritional ecology has been most extensively studied in terrestrial herbivorous mammals (Choat and Clements, 1998) and while progress has been made in other taxa, including marine herbivorous fishes (Clements *et al.*, 2009) and insects (Slansky, 1982; Simpson *et al.*, 2015), other groups such as predators are still lacking such information. Despite many papers citing nutritional deficiencies as a possible consequence of human interactions, few studies have actually investigated the proposed links (Jones and Reynolds, 2008), but nutritional stress is now being included in population modelling frameworks (National Academy of Sciences, Engineering and Medicine, 2016). However, we now have the tools to measure very detailed aspects of physiology related to nutrition (e.g. microbiomes: Lyons *et al.*, 2016; secondary compounds: Sotka and Whalen, 2008). Further work is clearly needed to identify the most pressing aspects of human-caused changes to food quality and quantity, such as: how animals choose which forage items to consume (e.g. can individuals learn about changes in toxicity or nutrient composition? Are genetic food preferences evolving in response to anthropogenic effects?); the interaction between energy availability and optimal digestion (Tracy *et al.*, 2006); the ways in which animals may plastically respond and/or evolve to cope with anthropogenic impacts (Crispo *et al.*, 2010; Sih *et al.*, 2011); how to design conservation solutions while recognizing that the choices animals make are constrained by evolutionary history (Schlaepfer *et al.*, 2002); and the frequency of synergistic effects across different anthropogenic impacts (Opdam and Wascher, 2004). A common pitfall of nutritional ecology and physiology is that hypotheses are often based on energetic intake and density (i.e. calories) rather than macronutrients and micronutrients, the latter of which we still know little about. We emphasize the importance of considering all aspects of nutrition (nutrient intake, foraging behaviour and digestive physiology, Fig. 1) when developing hypotheses about the effects of human activities on wildlife.

It is apparent that humans have altered many aspects of vertebrate nutrition. All anthropogenic impacts we focused on had documented negative effects on foraging behaviour and the availability of food, though most studies focused on quantity rather than quality of food. Very few investigated whether those changes affected digestion efficiency and energy acquisition, even though some forms of impact, such as provisioned food, logically seem like they should have large effects. In today’s changing world, animals eat food items they did not previously eat; they must invest more energy in foraging efforts than they previously had to (with the exception of wildlife that has access to human-provisioned food sources); and they now ingest more pollutants than they used to. All of these changes to nutritional intake can influence the reproductive capacity, growth and overall survival of wild animals. Our current understanding of the long-term effects of such modifications are poorly understood, and we urge for more research to consider the impacts that changing nutrition may have on animals in the long term as part of a broader conservation physiology approach (Cooke *et al.*, 2013). More specifically, the links between nutrient quality/quantity and various aspects of physiology (i.e. reproductive functions, immunity, stress response, etc.) and their population-level consequences should be investigated (National Academy of Sciences, Engineering and Medicine, 2016). By understanding the mechanisms by which nutrition is affected by anthropogenic factors, we may have a greater opportunity to minimize their threats.

## Funding

This work was supported by the Natural Sciences and Engineering Research Council of Canada [315 774-166] and the Canada Research Chairs program. D.R. is supported by Australian Research Council Linkage Grant [Project ID: LP 140100235].
